# Genetic diversity of the *Mycobacterium tuberculosis* Complex in San Luis Potosí, México

**DOI:** 10.1186/1756-0500-6-172

**Published:** 2013-05-01

**Authors:** Estela López-Rocha, Julio Juárez-Álvarez, Lina Riego-Ruiz, Leonor Enciso-Moreno, Francisco Ortega-Aguilar, Julián Hernández-Nieto, José A Enciso-Moreno, Rubén López-Revilla

**Affiliations:** 1 División de Biología Molecular, Instituto Potosino de Investigación Científica y Tecnológica, Camino a la Presa San José 2055, 78216 San Luis Potosí, SLP, Mexico; 2 Laboratorio Estatal de Salud Pública, Servicios de Salud del estado de San Luis Potosí, Begonias 180, 78399 San Luis Potosí, SLP, Mexico; 3 Unidad de Investigación Médica de Zacatecas, Instituto Mexicano del Seguro Social, Interior Alameda 45, 98000 Zacatecas, Zac, Mexico

**Keywords:** Tuberculosis, *Mycobacterium bovis*, Spoligotypes, Molecular epidemiology, San Luis Potosí, Mexico, Social determinants of tuberculosis

## Abstract

**Background:**

Although epidemiologic and socioeconomic criteria and biomedical risk factors indicate high-priority for tuberculosis (TB) control in Mexico, molecular epidemiology studies of the disease in the country are scarce.

**Methods:**

Complete sociodemographic and clinical data were obtained from 248 of the 432 pulmonary TB (PTB) cases confirmed from 2006 to 2010 on the population under epidemiological surveillance in the state of San Luis Potosí, México. From most PTB cases with complete data *Mycobacterium tuberculosis* complex (MTC) isolates were recovered and their spoligotypes, lineages and families, geographic distribution and drug resistance determined.

**Results:**

Pulmonary tuberculosis incidence ranged from 2.4 to 33.4 (cases per 100,000 inhabitants) in the six state sanitary jurisdictions that were grouped in regions of low (jurisdictions I-II-III), intermediate (jurisdictions IV-V) and high incidence (jurisdiction VI) with 6.2, 17.3 and 33.4 rates, respectively. Most patients were poor, 50-years-median-age males and housewives. Among the 237 MTC spoligotyped isolates, 232 corresponded to *M. tuberculosis* (104 spoligotypes in 24 clusters) and five to *M. bovis*. The predominant Euro-American lineage was distributed all over the state, the East-Asian lineage (Beijing family) in the capital city, the Indo-Oceanic (Manila family) in eastern localities, and *M. bovis* in rural localities.

**Conclusions:**

In San Luis Potosí TB affects mainly poor male adults and is caused by *M. tuberculosis* and to a minor extent by *M. bovis*. There is great genotypic diversity among *M. tuberculosis* strains, the Euro-American lineage being much more prevalent than the Indo-Oceanic and East-Asian lineages. The frequency of resistant strains is relatively low and not associated to any particular lineage.

## Background

In 2007 the global incidence of tuberculosis (TB) was 139 (cases per 100,000 inhabitants), whereas in the Americas it was 36.8 [1], and in Mexico 13.5 [2]. In the same year the Mexican states with highest incidences were Baja California (35.3) and Tamaulipas (32.7) whereas the incidence was 12.2 in the state of San Luis Potosí [2], where this study was performed. Although the national TB incidence is relatively low, the weight of epidemiologic and socioeconomic criteria and biomedical risk factors define Mexico as a high-priority country for TB control in the Americas [3].

TB reemergence, its association with the HIV-AIDS and diabetes epidemics [4,5] and the emergence and spread of MDR strains demand that epidemiological and genotyping data of *Mycobacterium tuberculosis* Complex (MTC) isolates be used to identify chains of transmission [6] and to differentiate TB cases due to endogenous reactivation [7].

Spoligotyping, based on the polymorphism of spacer sequences of the direct repeat region (DR) is used to differentiate MTC isolates [8]. Although less discriminatory than IS6110-based RFLP typing, it is a fast and cost-effective method allowing simultaneous analysis of numerous samples and generates contextual information on epidemiologically relevant MTC members [9]. Spoligotyping also identifies *M. bovis* strains, which usually carry few IS6110 copies [10].

The Euro-American lineage of *M. tuberculosis* predominates in Mexico [11], where some areas also have high frequencies of the Indo-Oceanic lineage [12]. In Mexico *M. bovis* also appears to be a relevant cause of pulmonary TB (PTB) in humans [13,14], and the Beijing family of the *M. tuberculosis* East-Asian lineage has been mentioned in a recent paper [5].

In this work we analyze the epidemiology, geographic distribution, lineages, families and drug resistance patterns of the MTC strains isolated from PTB cases in the state of San Luis Potosí, Mexico.

## Methods

### Territory and population

**Figure 1:**
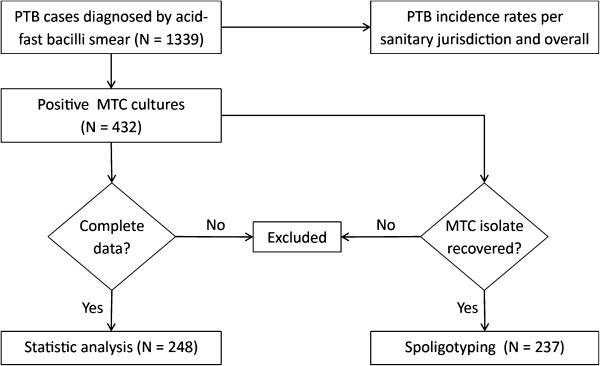
Flow diagram indicating the PTB cases, the cultured, recovered and spoligotyped MTC isolates, and the statistical, geographic and genotypic analyses performed for this study.

The state of San Luis Potosí, located in North-Central Mexico, is divided in 58 municipalities and six sanitary districts designated as jurisdictions I, II, III, IV, V and VI (Table 1). From January 2006 to March 2010, 1339 PTB cases were confirmed in the population submitted to passive epidemiologic surveillance (patients 15-years-old or older with productive cough for more than two weeks and positive acid-fast bacilli smear) and included in a DOT program whose scheme and drugs were provided and supervised by the State Tuberculosis Program, as defined by the Mexican Standard for Tuberculosis Prevention and Control [15]. Clinical information was elicited by medical personnel of dedicated brigades of the State TB Program and recorded in the National Epidemiologic Surveillance Platform. PTB incidence rates calculated from these cases were normalized for the population projected for 2010 [16] and complete sociodemographic and clinical data (name, sex, age, place of residence, occupation, formal education, previous TB history, contact with TB cases, concomitant disease and acid-fast bacilli smear) were collected from 248 cases (Figure 1).

**Table 1:** Population density, PTB incidence and MTC isolates per sanitary jurisdiction (January 2006 to March 2010)

**Sanitary jurisdiction**					Incidence rates and isolates analyzed*		
**Name**	**Municipalities**	**Area (km**^**2**^**)**	**Inhabitants**	**Inhabitants/km**^**2**^	**Cases (%)†**	**Incidence rate‡**	**MTC isolates (%)§**
I	2	1,724.1	1,054,522	611.6	352 (26.3)	7.9	39 (16.5)
II	11	21,757.9	216,348	9.9	28 (2.1)	3.0	8 (3.4)
III	13	13,447.0	273,705	20.4	28 (2.1)	2.4	3 (1.3)
IV	12	12,410.1	239,800	19.3	122 (9.1)	12.0	39 (16.5)
V	9	8,900.0	372,945	41.9	329 (24.6)	20.8	53 (22.4)
VI	11	2,306.8	338,193	146.6	480 (35.8)	33.4	95 (40.1)
Total	58	60,545.9	2,495,513	41.2	1339 (100.0)	12.6	237 (100.0)

*MTC, *Mycobacterium tuberculosis* complex.

†PTB cases confirmed by positive acid-fast bacilli smear.

‡PTB cases per 100,000 inhabitants.

§MTC cultures recovered and analyzed by spoligotyping.

Alcoholism was defined as a behavior disorder indicated by a rank > 8 in the Alcohol Use Disorders Identification Test (AUDIT) [17], and malnutrition by a body mass index < 18.5 [18]. Diabetes mellitus was defined by blood glucose levels > 126 mg/dl in fasting samples or > 200 mg/dl in random samples [19].

As indicator of socioeconomic level, the marginalization degree —a continuous integrative measure of the fraction of the population lacking access to goods and services essential for the development of basic capabilities— was stratified into five discrete indexes: very high, high, medium, low and very low [20]. The Ethics and Research Committee of the San Luis Potosí State Health Services approved the study.

### Culture and drug susceptibility of the MTC isolates

Sputum specimens were decontaminated with the Petroff method and simultaneously inoculated in the VersaTREK Myco System (TREK Diagnostic Systems, Cleveland, OH) and Lowenstein-Jensen medium. Positive cultures were identified as MTC members with the Cobas Amplicor *M. tuberculosis* test (Roche Diagnostics, Grenzach-Whylen, Germany). MTC isolates were cryopreserved at −70°C in Middlebrook 7H9 medium (TREK Diagnostic Systems, Cleveland, OH), recovered by reinoculation in the same medium and propagated in Lowenstein-Jensen medium from which colonies were picked and DNA extracted for genotyping.

Drug susceptibility was determined with the MYCO TB Susceptibility Testing kit (TREK Diagnostic Systems, Cleveland, OH), in which a standard dilution of each isolate was inoculated in Middlebrook 7H9 medium containing the drugs assayed at two concentrations: streptomycin 2 and 6 μg/ml; isoniazid 0.1 and 0.4 μg/ml; rifampicin 5 and 1 μg/ml; ethambutol 5 and 8 μg/ml. Each test was controlled with the *M. tuberculosis* H37Rv sensitive strain and the ATCC 35820, 35822, 35837 and 35838 strains as controls for streptomycin-, isoniazid-, ethambutol-, and rifampicin-resistance, respectively. To validate each assay, resistant cultures were compared with drug-free controls.

### Spoligotyping

From 432 MTC strains isolated at the Public Health State Laboratory, 237 (54.9%) were sampled by convenience (Figure 1): 39 from jurisdiction I, eight from jurisdiction II, three from jurisdiction III, 39 from jurisdiction IV, 53 from jurisdiction V and 95 from jurisdiction VI. Sample size was approximately proportional to the number of PTB cases recorded in each jurisdiction (Table 1). The MTC isolates selected were those maintained by subculture that yielded enough DNA for genotyping.

MTC DNA was extracted with the cetyl trimethylammonium bromide (CTAB) method [21]. Spoligotyping was carried out according to the manufacturer’s instructions with the Isogen kit which includes a nitrocellulose membrane with 43 immobilized spacer sequences of the direct repeat (DR) region (Life Science, Maarssen, Netherlands). The spacers were amplified using primers DRa (5′-GGTTTTGGGTCTGACGAC-3′) and DRb (5′-CCGAGAGGGGACGGAAAC-3′) [8]. *M. tuberculosis* H37Rv and CDC1551 and *M. bovis* BCG DNAs were used as positive controls. Hybridization of the PCR products was detected with the Direct Nucleic Acid Labelling and Detection System (Amersham International plc, Buckinghamshire, United Kingdom). Spoligotypes were identified with the online MIRU-VTRN*plus* application [22]. MTC lineages and families were assigned after identifying the best matches among genotypes from the internal reference database with a categorical coefficient of 1.7. For additional tree-based identification, a dendrogram of spoligotype patterns was generated using the un-weighted pair group method with the neighbor-joining algorithm. Spoligotype patterns were also compared with those in the SITVIT2 database (Institut Pasteur de Guadeloupe, http://www.pasteur-guadeloupe.fr:8081/SITVITDemo/) [23] and the Mbovis.org database (Veterinary Laboratories Agency, http://www.mbovis.org/index.php).

### Statistical analysis and geographic distribution of MTC species, lineages and families

Statistical analysis was carried out with the SPSS 18 software (IBM Corporation, Somers, NY). The Pearson χ^2^ test was used to assess differences in sociodemographic and clinical variables among geographic zones, lineages and drug resistance. Differences in the ages of cases among geographic zones were assessed by one-way ANOVA. P values ≤ 0.05 were considered statistically significant. PTB cases and MTC lineages and families were linked to geographical coordinates provided by the Instituto Nacional de Estadística Geografía e Informática at city and community scale [24]. MTC species, lineages and families were mapped using ArcMap 9.2 software (Esri, Redlands, CA).

## Results

### Pulmonary TB incidence per sanitary jurisdiction and region

From January 2006 to March 2010 the PTB incidence rate for the state of San Luis Potosí was 12.6, with remarkable differences per jurisdiction.

**Figure 2:**
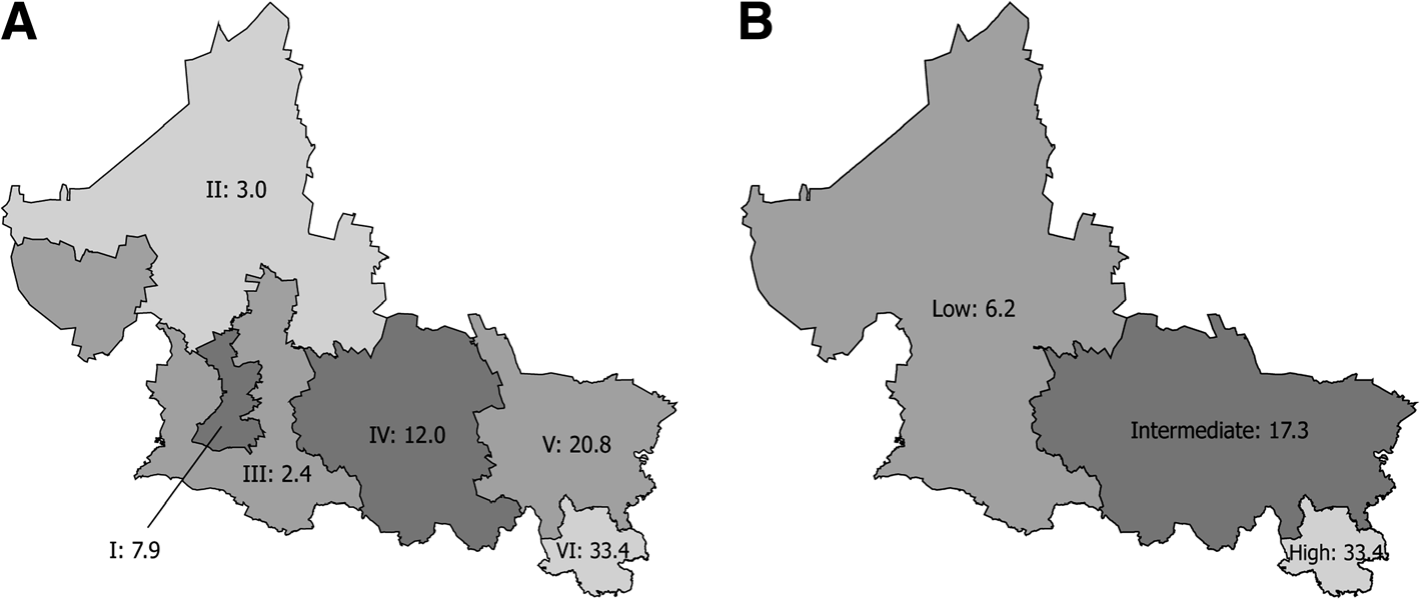
**PTB incidence rates by jurisdiction and region.** (**A**) Incidence rate by sanitary jurisdiction. Roman numerals followed by Arabic numerals indicate each one of the six sanitary jurisdictions of the state of San Luis Potosí and their corresponding PTB incidence rates (per 100,000 inhabitants) in 2008. (**B**) Regions of low (jurisdictions I, II and III), intermediate (jurisdictions IV and V), and high (Jurisdiction VI) incidence, with their corresponding rates.

Jurisdictions I, II and III had lower incidence rates (7.9, 3.0, and 2.4, respectively); jurisdictions IV and V had intermediate rates (12.0 and 20.8, respectively), and jurisdiction VI, located in the southeastern end of the state, had the maximum rate (33.4). The combined incidence rate of the three jurisdictions with higher rates (IV, V and VI) was almost four times above that of jurisdictions with the lower rates (I, II and III) (Figure 2A).

In view of these differences we divided the state in three regions: the low incidence region (jurisdictions I, II and III with a combined rate of 6.2); the intermediate region (jurisdictions IV and V with a combined rate of 17.3); and the high incidence region (jurisdiction VI, with a rate of 33.4) (Figure 2B, Table 2).

**Table 2:** Sociodemographic and clinical variables of the PTB cases in the low-, intermediate- and high-incidence regions of the state

**Variable**	Incidence region*				**P†**
	**Low (6.2)**	**Intermediate (17.3)**	**High (33.4)**	**Overall (12.6)**	
Number of PTB cases	63	83	102	248	---
Sex					
Male	40 (63.5) ‡	47 (56.6)	68 (66.7)	155 (62.5)	0.670
Female	23 (36.5)	36 (43.4)	34 (33.3)	93 (37.5)	0.861
Median age					
Years (range)	45 (18–86)	48 (15–80)	55 (16–95)	50 (15–95)	0.031
Occupation					
Farm worker	15 (23.8)	25 (30.1)	43 (42.2)	83 (33.5)	0.116
Housewife	18 (28.6)	31 (37.3)	28 (27.5)	77 (31.0)	0.449
Unemployed	11 (17.5)	12 (14.5)	19 (18.6)	42 (16.9)	0.779
Employee	12 (19.0)	4 (4.8)	3 (2.9)	19 (7.7)	0.001
Independent	5 (7.9)	8 (9.6)	7 (6.9)	20 (8.1)	0.819
Another	2 (3.2)	3 (3.6)	2 (2.0)	7 (2.8)	0.779
Formal education					
None	13 (20.6)	21 (25.3)	36 (35.3)	70 (28.2)	0.522
Incomplete primary	21 (33.3)	29 (34.9)	33 (32.4)	83 (33.5)	0.638
Primary	16 (25.4)	21 (25.3)	20 (19.6)	57 (23.0)	0.951
Secondary	11 (17.5)	12 (14.5)	11 (10.8)	34 (13.7)	0.192
College	2 (3.2)	0 (0)	2 (2.0)	4 (1.6)	0.301
Habitat					
Rural	11 (17.5)	45 (54.2)	88 (86.3)	144 (58.1)	< 0.001
Urban	52 (82.5)	38 (45.8)	14 (13.7)	104 (41.9)	< 0.001
Marginalization index					
Very high	0 (0)	9 (10.8)	0 (0)	9 (3.6)	< 0.001
High	12 (19)	18 (21.7)	102 (100.0)	132 (53.2)	< 0.001
Medium	2 (3.2)	33 (39.8)	0 (0)	35 (14.1)	< 0.001
Low	1 (1.6)	23 (27.7)	0 (0)	24 (9.7)	< 0.001
Very low	48 (76.2)	0 (0)	0 (0)	48 (19.4)	< 0.001
Previous history of TB					
No	59 (93.7)	75 (90.4)	90 (88.2)	224 (90.3)	0.951
Yes	4 (6.3)	8 (9.6)	12 (11.8)	24 (9.7)	0.549
Contact with TB cases					
Yes	8 (12.7)	24 (28.9)	29 (28.4)	61 (24.6)	0.086
No	55 (87.3)	59 (71.1)	73 (28.4)	187 (75.4)	0.449
Concomitant disease					
None	34 (54.0)	37 (44.6)	50 (49.0)	121 (48.8)	0.741
Diabetes	20 (31.7)	19 (22.9)	14 (13.7)	53 (21.4)	0.047
Malnutrition	4 (6.3)	15 (18.1)	22 (21.6)	41 (16.5)	0.061
Alcoholism	1 (1.6)	7 (8.4)	15 (14.7)	23 (9.3)	0.026
HIV-AIDS	4 (6.3)	1 (1.2)	0 (0)	5 (2.0)	0.017
Other	0 (0)	4 (4.8)	1 (1.0)	5 (2.0)	0.082
Acid-fast bacilli smear					
Positive	56 (88.9)	78 (94.0)	100 (98.0)	234 (94.4)	0.016
Negative	7 (11.1)	5 (6.0)	2 (2.0)	14 (5.6)	0.584

*Number of cases per 100,000 inhabitants in parenthesis.

†*χ*^2^. One-way ANOVA, for age difference.

‡Numbers in parenthesis are percentages between variable categories, except in the median age where they are ranges.

In the high incidence region the median age of confirmed PTB cases (55 years) was ten years higher and significantly different (P = 0.031) to that of the low incidence region, and seven years higher than that of the intermediate incidence region. The proportion of patients living in rural communities increased significantly (P < 0.001) from the low (17.5%) to the intermediate (54.2%) and high incidence (86.3%) regions. The proportion of patients residing in municipalities of high marginalization also increased significantly (P < 0.001) from the low (19.0%) to the intermediate (21.7%) and high incidence (100.0%) regions.

### Features of the PTB cases

The overall median age of PTB cases in the state was 50 years. Two thirds (62.5%) were men, one half (50.4%) were unemployed and employed construction workers or farm workers, and one third (31.0%) were housewives. Nearly two thirds (61.7%) had either incomplete primary education or no formal education at all. More than half (58.1%) resided in rural localities and 53.2% in highly marginalized municipalities (Table 2).

Most cases (90.3%) did not have a previous history of TB and 75.0% declared not to have contacted other PTB cases. One half (51.2%) had a concomitant disease; the most frequent was diabetes (21.4%), followed by malnutrition (16.5%) and alcoholism (9.3%).

Diabetes-PTB association decreased significantly (P = 0.047) from the low (31.7%) to the intermediate (22.9%) and high incidence (13.7%) regions. In contrast, malnutrition-PTB association did not increase significantly (P = 0.061) from the high incidence region (6.3%) to the intermediate (18.1%) and low incidence (21.6%) regions. Alcoholism as a concomitant disease increased significantly (P = 0.026) from the low incidence region (1.6%) to the intermediate (8.4%) and high incidence (14.7%) regions. Marginalization is directly related to malnutrition and alcoholism and inversely related to diabetes since the first two concomitant diseases predominate in the high incidence region and diabetes in the low incidence region.

### Lineages, families and clusters of the MTC isolates

**Figure 3:**
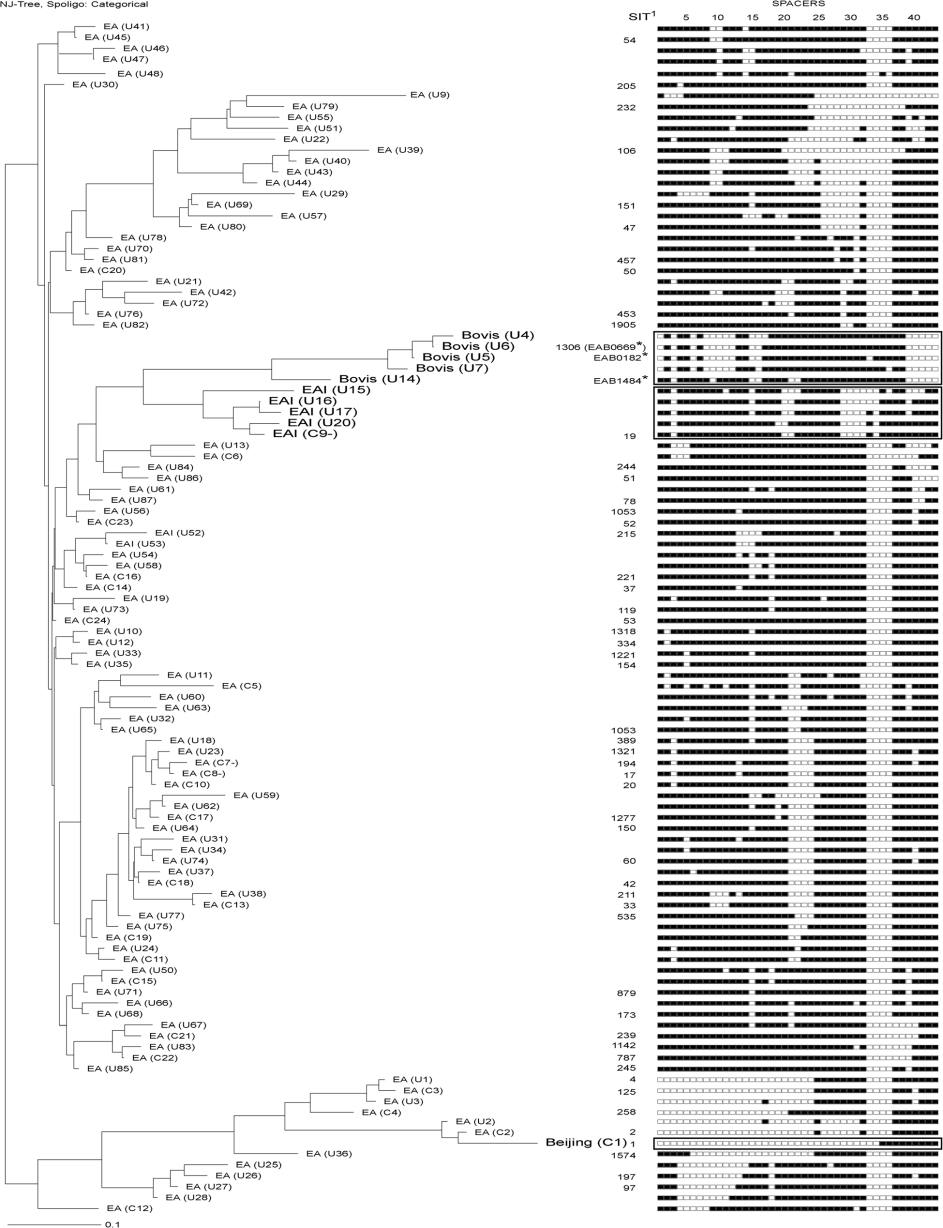
**Dendrogram, families and spoligotype patterns of the 109 MTC genotypes identified.***M. tuberculosis* isolates of the Euro-American lineage are indicated with smaller font labels and those of other lineages and *M. bovis* isolates are indicated with larger font labels and their spoligotype patterns enclosed in rectangles. SIT: Spoligo international type numbers from the SpolDB4 database. *M. bovis* spoligotype identifiers (marked with asterisks) from the Mbovis.org database.

Spoligotypes of the 237 isolates analyzed corresponded to two MTC species: 232 to *M. tuberculosis* (97.8%) and five to *M*. *bovis* (2.1%). Four lineages and 109 genotypes were identified (Figure 3). Fifty five genotypes (50.5%) had already been registered (53 in SpolDB4 and three in the Mbovis.org database; one of the five *M. bovis* genotypes had been registered in both databases).

Of the *M. tuberculosis* isolates, 221 (95.3%) had Euro-American lineage spoligotypes, among which 118 (53.4%) were SIT53, SIT42, SIT20, SIT221, SIT239, SIT50, SIT787, SIT17 and SIT258, and 55 isolates (24.9%) had previously undetected (“orphan”) Euro-American genotypes. Eleven isolates had spoligotypes characteristic of other lineages and families: eight (3.4%) corresponded to the EAI-Manila family of the Indo-Oceanic lineage, and three isolates with identical spoligotypes (1.3%) to the Beijing family of the East-Asian lineage. Of the 109 genotypes identified, 85 (78.0%) appeared in a single isolate and the remaining 24 (22.0%) in clusters of two up to 68 isolates (Table 3).

**Table 3:** Spoligotype patterns and octal codes of the 24 MTC clusters found

**Cluster ID**	SIT*	**N†**	**Spoligotype pattern**	**Octal code**
c-24	53	68	1111111111111111111111111111111100001111111	777777777760771
c-18	42	14	1111111111111111111100001111111100001111111	777777607760771
c-10	20	7	1101111111111111111100001111111100001111111	677777607760771
c-16	221	7	1111111111111101101111111111111100001111111	777766777760771
c-21	239	6	1111111111111111111111111111111100000000111	777777777760031
c-20	50	5	1111111111111111111111111111110100001111111	777777777720771
c-22	787	5	1111111111111111111111111111111100000001111	777777777760071
c-9	19	4	1101111111111111111001111111000010111111111	677777477413771
c-12	2111	3	1110000011111111111111111111110100001111111	701777777720771
c-1	1	3	0000000000000000000000000000000000111111111	000000000003771
c-4	258	3	0000000000000000000011111111111100001111111	000000177760771
c-8	17	3	1101111111110111111100001111111100001111111	677737607760771
c-11	---	2	1101111111111111111100111111111100001111111	677777637760771
c-13	33	2	1111111100011111111100001111111100001111111	776177607760771
c-14	37	2	1111111111110111111111111111111100001111111	777737777760771
c-15	---	2	1111111111111101101111111111111100001101111	777766777760671
c-17	1277	2	1111111111111111110100001111111100001111111	777777207760771
c-19	2070	2	1111111111111111111100111111111100001111111	777777637760771
c-23	52	2	1111111111111111111111111111111100001110111	777777777760731
c-2	2	2	0000000000000000000000001000000100001111111	000000004020771
c-3	125	2	0000000000000000000000001111111100001110111	000000007760731
c-6	---	2	1100011111111111111111111111111100000000111	617777777760031
c-7	194	2	1101111111110111111100001111111100001110111	677737607760731
c-5	---	2	1011011011011101111100111111111000001101111	555567637740671

*SIT, Spoligo international type number in SpolDB4.

†Number of isolates sharing the same spoligotype.

One hundred and fifty two isolates (64.1%) were grouped in clusters. The two largest clusters were formed by 68 isolates of SIT53 genotype and 14 isolates of SIT42 genotype (Table 3).

**Figure 4:**
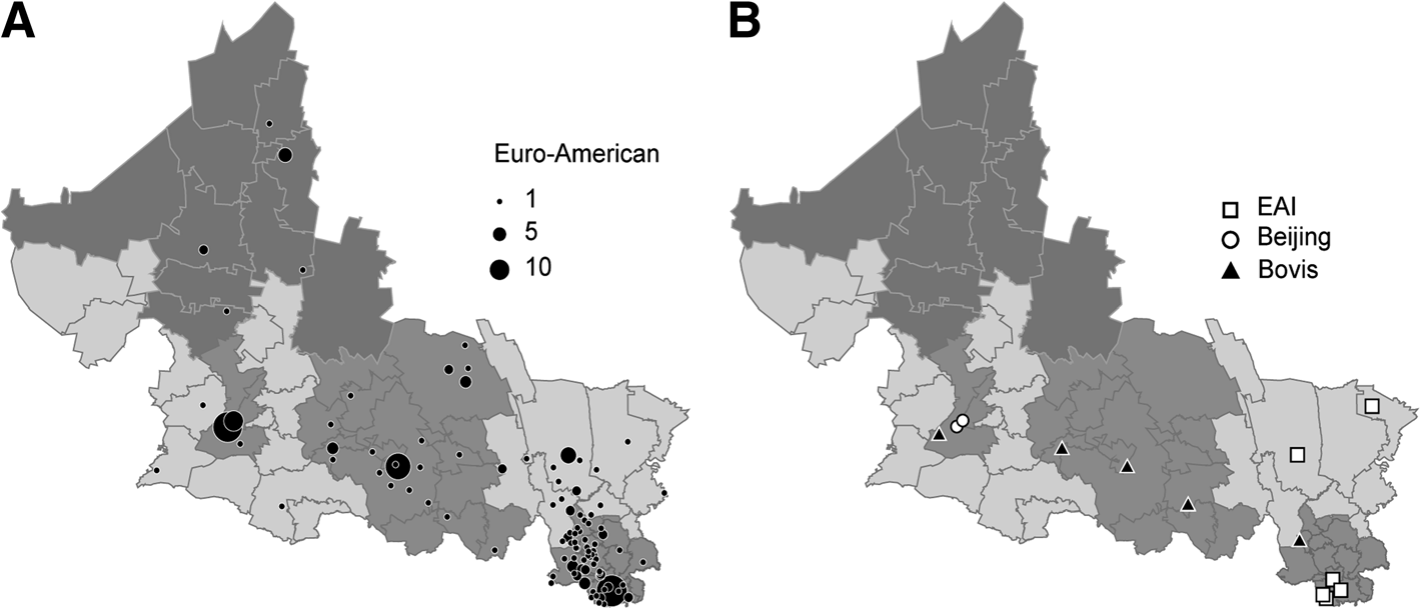
**Geographic distribution of the Euro-American and other MTC lineages found.** (**A**) Distribution of the Euro-American lineage. (**B**) Distribution of the EAI, Beijing and Bovis lineages. The symbols indicate the localities with PTB cases associated to each lineage; the area of each circle is proportional to the number of cases per locality.

Euro-American isolates were distributed all over the state (Figure 4A). Beijing isolates were located in the San Luis Potosí City metropolitan area (Figure 4B), EAI-Manila isolates in the easternmost jurisdictions V and VI, and *M. bovis* isolates in five southern rural communities from jurisdictions I, IV and V (Figure 4B).

Thus in the state of San Luis Potosí there is great genetic diversity in the circulating MTC strains, among which the Euro-American lineage predominates.

### Lineages and families of drug-resistant MTC isolates

Twenty-three of the 237 spoligotyped isolates were resistant to one or more drugs: nine were resistant to isoniazid, two to rifampicin, two to other drugs, and 10 were MDR (Table 4).

**Table 4:** Lineages of the drug-resistant MTC isolates

**Lineage**	**Single-drug resistance**			**MDR**	**Total**
	**Isoniazid**	**Rifampicin**	Other drugs*		
Euro-American	8 (3.4%)	1 (0.4%)	2 (0.8%)	9 (3.8%)	20 (8.4%)
Indo-Oceanic (EAI)	0	1 (0.4%)	0	1 (0.4%)	2 (0.8%)
East-Asian (Beijing)	1 (0.4%)	0	0	0	1 (0.4%)
Total	9 (3.8)	2 (0.8%)	2 (0.8%)	10 (4.2%)	23 (9.7%)

*Isolates resistant to one first line drug other than isoniazid or rifampicin.

Eleven of the 13 single-drug-resistant isolates were of the Euro-American lineage. Among the MDR isolates, nine were of the Euro-American lineage and one was of the Indo-Oceanic (EAI-Manila) lineage. One rifampicin-resistant isolate was of the EAI-Manila family and one isoniazid-resistant isolate was of the Beijing family. Thirteen single drug-resistant and eight MDR isolates came from jurisdictions V and VI.

In summary, 9.7% of the isolates were resistant to one or more drugs and 4.2% were MDR; 3.8% were resistant only to isoniazid, 0.8% only to rifampicin and 0.8% to other drugs. Although most (84.6%) of the single drug-resistant isolates and 90% of the MDR ones were of the Euro-American lineage, association between drug resistance of any kind with the lineage or family of the isolates was not significant (P = 0.653).

## Discussion

Low socioeconomic status, a TB determinant, includes risk factors such as food insecurity, poor housing and cultural, financial and geographic barriers for access to health services [25]. We confirmed that in San Luis Potosí PTB incidence correlates with low socioeconomic status since nearly two thirds of the patients resided in rural communities and more than half of them lived in highly marginalized municipalities.

Overall PTB incidence in the state (12.6) is remarkably different to that of each sanitary jurisdiction and led us to divide the state in three incidence regions (Figure 2). The low incidence region comprises jurisdictions I, II and III; the intermediate incidence region comprises jurisdictions IV and V. The high incidence region corresponds to jurisdiction VI; located in the southeastern part of the state, it is a tropical, predominantly rural and highly marginalized area, with 48% of indigenous population [21] and a population density 1.5 higher than that of the other jurisdictions combined, except for jurisdiction I, where the capital city is located [16].

Marginalization indexes differentiate municipalities by the impact of variables threatening life quality such as house overcrowding, low educational level and lack of electricity, running water and sanitary services [20]. In San Luis Potosí we confirmed that regional TB incidence correlates with the socioeconomic gradient, as is known to occur within countries and within communities, the poorest ones being affected most [26].

Although residents of urban areas are believed to be at higher risk of TB [25], most of the cases in the high incidence region reside in rural communities. This finding may be due to risk factors such as overcrowding, indoor pollution, and poor ventilation in the rural communities affected. Marginalization is directly related to the incidence of malnutrition and alcoholism and inversely related to the incidence of diabetes since the first two concomitant diseases predominate in the high incidence region and diabetes in the low incidence region.

Sociodemographic and clinical variables in the low incidence region are similar to those in Monterrey and Acapulco [11,12], two Mexican cities of relatively low marginalization. In contrast, such variables in the high incidence region resemble more those of highly marginal rural regions of the Mexican state of Chiapas [27] where the average age of PTB cases is around 40 years, whereas in the high incidence region of San Luis Potosí it is close to 53 years. Both the transmission-mutation index derived from the spoligoforest analysis and the transmission chains identified by MIRU-VNTR genotyping indicate that recent transmission contributes marginally to the epidemiology of TB (E. López-Rocha et al., manuscript in preparation), whereas the advanced age of the cases and the high proportion of positive acid-fast bacilli smears (94.4%) indicates delayed disease detection. These findings support the notion that in San Luis Potosí endogenous reactivation contributes to TB epidemiology much more than recent transmission [28].

One tenth of the MTC isolates in San Luis Potosí are resistant to one or more drugs; 4.2% are MDR, a proportion below the national average of 5.8% [29] and lower than that of the American continent (7.2%) [3]. No statistically significant association of drug resistance and MTC lineage was found.

Most of the spoligotyped MTC isolates corresponded to *M. tuberculosis* and only 2.1% to *M. bovis*. *M. tuberculosis* isolates had 104 different genotypes among which only 52 (50.0%) had been registered in SpolDB4, and correspond to three lineages: Euro-American, Indo-Oceanic and East-Asian. A comprehensive study of MTC genotypes based on the analysis of large sequence polymorphisms found that Euro-American is the predominant *M. tuberculosis* lineage in Latin America [30]. The same lineage has been shown to be prevalent in the Mexican cities of Orizaba [5] and Monterrey [11], and we also found it to be the most prevalent in San Luis Potosí (95.3%) with SIT53, SIT42, SIT20, SIT221, SIT239, SIT50, SIT787, SIT17 and SIT258 as its main genotypes.

In San Luis Potosí 21.8% of the *M. tuberculosis* genotypes are grouped in clusters, the largest one being that with 68 isolates of the SIT53 genotype, and the second largest with 14 isolates of the SIT42 genotype. The SIT53 genotype, belonging to the ill-defined T1 sublineage, is ubiquitous and the most frequent in SpolDB4 [23]; the SIT42 genotype is also ubiquitous and ranks sixth in the same database. In the Mexican city of Monterrey the largest cluster corresponds to the SIT53.

The least frequent *M. tuberculosis* lineages in San Luis Potosí are the Indo-Oceanic (EAI-Manila family, 3.4%) and the East-Asian (Beijing family, 1.3%). The EAI-Manila family —endemic in Southeast Asia, South India and East Africa— was recognized in Filipino immigrants in the United States and later shown to be highly prevalent in the Philippines [31]. It was identified in Mexico for the first time in Orizaba, Veracruz [32]. Four of our eight EAI isolates correspond to the ubiquitous and common SIT19 genotype and could also have originated from the Philippines, given the commercial links of that region with Mexico from 1565 to 1815 through the Manila-Acapulco galleon [33]. This hypothesis is consistent with the recent finding that one fourth of the *M. tuberculosis* isolates in Acapulco correspond to the EAI family [12], where the largest cluster has the SIT19 genotype previously found in Monterrey [11], and now by us in a cluster with four isolates.

Our three Beijing isolates have the SIT1 genotype and, although a transmission chain cannot be discarded, their low prevalence suggests they arose from independent transmission events. SIT1, the second most frequent genotype in SpolDB4 [23], is ubiquitous —it has been identified in 29 countries and eight geographic areas—, epidemic (propagation index = 44.2), highly virulent [34] and appears to have selective advantages over other genotypes [35]. Only one Mexican PTB case by a Beijing strain from Orizaba, Veracruz, has been published [5]. Although the presence of this genotype in San Luis Potosí is worrying, its low frequency suggests that up to now its transmission is negligible.

The genetic diversity of the MTC appears to be geographically structured [30], since some strains preferably infect certain human populations and the association between pathogens and their hosts appear to be stable [36]. These data led to the notion that the worldwide TB pandemic is the sum of genetically different outbreaks [23]. Since the Euro-American lineage is distributed all over the state, the Beijing family is limited to the capital metropolitan area and the EAI-Manila family to the eastern part of the state, the TB epidemic in San Luis Potosí may be interpreted as a part of the wider TB epidemic in Latin America, with an outbreak of the EAI lineage and some Beijing cases.

All our five *M. bovis* isolates have different spoligotypes and come from rural communities located in the dairy region of the state. The prevalence of human TB of bovine origin in San Luis Potosí may be much higher than 2.1% since bovine TB has been eradicated from only one Mexican state [37], and all the MTC isolates recovered for this study were initially cultured in Lowenstein-Jensen medium, more appropriate for *M. tuberculosis* than for *M. bovis*[38]. Therefore, the surveillance of human TB caused by *M. bovis* must be reinforced in the state health program.

The MTC strains included in this study were only those that were kept and recovered at the Public Health State Laboratory, where most state TB cases are concentrated. Although scarce, cases from individuals affiliated to the Mexican Social Security Institute or other organizations may have been overlooked. On the other hand, minor mistakes in the assignment of MTC lineages and families may have occurred, since the dendrogram-based similarities used by us to identify the isolates are known to be 94.1% accurate [39].

The prevalence of human TB due to *M. bovis* correlates with TB prevalence in cattle, and most human cases are related to consumption or handling of contaminated dairy products [40]. Although the prevalence of human TB of bovine origin in Mexico is unknown, 5.3% of the MTC strains isolated from sputum samples of PTB cases in a zone endemic for bovine TB in the Mexican state of Querétaro had *M. bovis* spoligotypes [13]. In the same endemic zone a study on asymptomatic farm workers identified *M. bovis* spoligotypes in 10.8% of sputum and 5.3% of urine samples [14]. Other population studies in Mexico found *M. bovis* spoligotype frequencies of 0.6% in Monterrey [11] and 0.4% in Acapulco [12].

## Conclusions

This is the first study on the molecular epidemiology of TB which covers a complete state in Mexico. In the state of San Luis Potosí, PTB incidence correlates with low socioeconomic status and the prevalent MTC strains show high genetic diversity and appear to be part of the wider TB epidemic in Latin America. Our findings show the need to implement TB control plans adapted to each incidence region in the state, including routine monitoring of the circulating MTC genotypes and reinforcing *M. bovis* surveillance.

## Competing interest

The authors declare that they have no competing interests.

## Authors’ contributions

LRR, RLR, AE and ELR conceived and designed the study. ELR participated in the research grant application and molecular typing, LE in molecular typing, FOA and JHN in culturing and drug-susceptibility testing of the MTC isolates, and JJA in epidemiological and molecular data gathering and analysis. ELR, JJA and RLR prepared the first draft of the manuscript. All authors read the manuscript, participated in its editing and approved the final version.

## References

[B1] World Health Organization Global Tuberculosis Control: Epidemiology, Strategy, Financing 2009 Geneva, Switzerland: WHO

[B2] Tuberculosis in Mexico [in Spanish] [http://www.dgepi.salud.gob.mx/boletin/2008/2008_PDF/sem14.pdf]

[B3] Panamerican Health Organization Tuberculosis in the Americas. 2008 Regional Report [in Spanish] 2008 Washington, DC: The Organization

[B4] Ponce-De-Leon A Garcia-Garcia Md Mde L Garcia-Sancho MC Gomez-Perez FJ Valdespino-Gomez JL Olaiz-Fernandez G Rojas R Ferreyra-Reyes L Cano-Arellano B Bobadilla M Tuberculosis and diabetes in southern Mexico Diabetes Care 2004 27 1584 1590 15220232 10.2337/diacare.27.7.1584

[B5] Jimenez-Corona ME Garcia-Garcia L Leon AP Bobadilla-del Valle M Torres M Canizales-Quintero S Palacios-Merino C Molina-Hernandez S Martinez-Gamboa RA Juarez-Sandino L Research on conventional and molecular epidemiology of tuberculosis in Orizaba, Veracruz, 1995–2008 [in Spanish] Salud Publica Mex 2009 51 Suppl 3 S470 S478 20464221

[B6] van der Spuy GD Warren RM Richardson M Beyers N Behr MA van Helden PD Use of genetic distance as a measure of ongoing transmission of Mycobacterium tuberculosis J Clin Microbiol 2003 41 5640 5644 14662954 10.1128/JCM.41.12.5640-5644.2003 PMC308980

[B7] van Rie A Warren R Richardson M Victor TC Gie RP Enarson DA Beyers N van Helden PD Exogenous reinfection as a cause of recurrent tuberculosis after curative treatment N Engl J Med 1999 341 1174 1179 10519895 10.1056/NEJM199910143411602

[B8] Kamerbeek J Schouls L Kolk A van Agterveld M van Soolingen D Kuijper S Bunschoten A Molhuizen H Shaw R Goyal M van Embden J Simultaneous detection and strain differentiation of Mycobacterium tuberculosis for diagnosis and epidemiology J Clin Microbiol 1997 35 907 914 9157152 10.1128/jcm.35.4.907-914.1997 PMC229700

[B9] Filliol I Driscoll JR van Soolingen D Kreiswirth BN Kremer K Valetudie G Dang DA Barlow R Banerjee D Bifani PJ Snapshot of moving and expanding clones of Mycobacterium tuberculosis and their global distribution assessed by spoligotyping in an international study J Clin Microbiol 2003 41 1963 1970 12734235 10.1128/JCM.41.5.1963-1970.2003 PMC154710

[B10] van Soolingen D de Haas PE Hermans PW Groenen PM van Embden JD Comparison of various repetitive DNA elements as genetic markers for strain differentiation and epidemiology of Mycobacterium tuberculosis J Clin Microbiol 1993 31 1987 1995 7690367 10.1128/jcm.31.8.1987-1995.1993 PMC265684

[B11] Molina-Torres CA Moreno-Torres E Ocampo-Candiani J Rendon A Blackwood K Kremer K Rastogi N Welsh O Vera-Cabrera L Mycobacterium tuberculosis spoligotypes in Monterrey, Mexico J Clin Microbiol 2010 48 448 455 19940048 10.1128/JCM.01894-09 PMC2815641

[B12] Nava-Aguilera E Lopez-Vidal Y Harris E Morales-Perez A Mitchell S Flores-Moreno M Villegas-Arrizon A Legorreta-Soberanis J Ledogar R Andersson N Clustering of Mycobacterium tuberculosis cases in Acapulco: Spoligotyping and risk factors Clin Dev Immunol 2011 2011 408375 21197077 10.1155/2011/408375 PMC3004385

[B13] Perez-Guerrero L Milian-Suazo F Arriaga-Diaz C Romero-Torres C Escartin-Chavez M Molecular epidemiology of cattle and human tuberculosis in Mexico [in Spanish] Salud Publica Mex 2008 50 286 291 18670719 10.1590/s0036-36342008000400006

[B14] Milian-Suazo F Perez-Guerrero L Arriaga-Diaz C Escartin-Chavez M Molecular epidemiology of human cases of tuberculosis by Mycobacterium bovis in Mexico Prev Vet Med 2010 97 37 44 20828845 10.1016/j.prevetmed.2010.06.015

[B15] Ministry of Health Modification to the Official Mexican Standard NOM-006-SSA2-1993, for the prevention and control of tuberculosis in primary health attention [in Spanish] Book Modification to the Official Mexican Standard NOM-006-SSA2-1993, for the prevention and control of tuberculosis in primary health attention [in Spanish] 2005 Mexico City: The Ministry 1 12

[B16] Mexico Population Projections: 2005–2030 [in Spanish] [http://www.conapo.gob.mx/work/models/CONAPO/Resource/136/1/images/municipales.xls]

[B17] Babor T Higgins-Biddle J Saunders J Monteiro M The alcohol use disorders identification test. Guidelines for Use in Primary Care 2001 Geneva, Switzerland: World Health Organization

[B18] World Health Organization Obesity: Preventing and Managing the Global Epidemic. Report of a WHO Consultation 2000 Geneva, Switzerland: WHO 11234459

[B19] The Expert Committee on the Diagnosis and Classification of Diabetes Mellitus Report of the Expert Committee on the Diagnosis and Classification of Diabetes Mellitus Diabetes Care 2000 23 Suppl 1 S4 S19 12017675

[B20] Methodology for Estimation of the Marginalization Index [in Spanish] [http://www.conapo.gob.mx/index.php?option=com_content&view=article&id=126&Itemid=293]

[B21] Honore-Bouakline S Vincensini JP Giacuzzo V Lagrange PH Herrmann JL Rapid diagnosis of extrapulmonary tuberculosis by PCR: impact of sample preparation and DNA extraction J Clin Microbiol 2003 41 2323 2329 12791844 10.1128/JCM.41.6.2323-2329.2003 PMC156509

[B22] Weniger T Krawczyk J Supply P Niemann S Harmsen D MIRU-VNTRplus: a web tool for polyphasic genotyping of Mycobacterium tuberculosis complex bacteria Nucleic Acids Res 2010 38 Suppl W326 W331 20457747 10.1093/nar/gkq351 PMC2896200

[B23] Brudey K Driscoll JR Rigouts L Prodinger WM Gori A Al-Hajoj SA Allix C Aristimuno L Arora J Baumanis V Mycobacterium tuberculosis complex genetic diversity: mining the fourth international spoligotyping database (SpolDB4) for classification, population genetics and epidemiology BMC Microbiol 2006 6 23 16519816 10.1186/1471-2180-6-23 PMC1468417

[B24] Vectorial Data Set of the National Geostatistical Frame [in Spanish] [http://www.inegi.org.mx/geo/contenidos/geoestadistica/marco_geoestadístico.aspx]

[B25] Hargreaves JR Boccia D Evans CA Adato M Petticrew M Porter JD The social determinants of tuberculosis: from evidence to action Am J Public Health 2011 101 654 662 21330583 10.2105/AJPH.2010.199505 PMC3052350

[B26] Lonnroth K Jaramillo E Williams BG Dye C Raviglione M Drivers of tuberculosis epidemics: the role of risk factors and social determinants Soc Sci Med 2009 68 2240 2246 19394122 10.1016/j.socscimed.2009.03.041

[B27] Sanchez-Perez HJ Diaz-Vazquez A Najera-Ortiz JC Balandrano S Martin-Mateo M Multidrug-resistant pulmonary tuberculosis in Los Altos, Selva and Norte regions, Chiapas, Mexico Int J Tuberc Lung Dis 2010 14 34 39 20003692

[B28] Borgdorff MW Nagelkerke NJ de Haas PE van Soolingen D Transmission of Mycobacterium tuberculosis depending on the age and sex of source cases Am J Epidemiol 2001 154 934 943 11700248 10.1093/aje/154.10.934

[B29] World Health Organization Anti-Tuberculosis Drug Resistance in the World 2008 Geneva: The Organization

[B30] Gagneux S DeRiemer K Van T Kato-Maeda M de Jong BC Narayanan S Nicol M Niemann S Kremer K Gutierrez MC Variable host-pathogen compatibility in Mycobacterium tuberculosis Proc Natl Acad Sci USA 2006 103 2869 2873 16477032 10.1073/pnas.0511240103 PMC1413851

[B31] Douglas JT Qian L Montoya JC Musser JM Van Embden JD Van Soolingen D Kremer K Characterization of the Manila family of Mycobacterium tuberculosis J Clin Microbiol 2003 41 2723 2726 12791915 10.1128/JCM.41.6.2723-2726.2003 PMC156522

[B32] Martinez-Gamboa A Ponce-de-Leon A Galindo-Fraga A Bobadilla-del-Valle M Kato-Maeda M Robertson BD Young DB Small PM Sifuentes-Osornio J Molecular analysis of Mycobacterium tuberculosis strains with an intact pks15/1 gene in a rural community of Mexico Arch Med Res 2008 39 809 814 18996296 10.1016/j.arcmed.2008.08.006

[B33] Schurz WL The Manila galleon and California Southwestern Historical Quaterly 1917 21 107 126

[B34] Hanekom M van der Spuy GD Streicher E Ndabambi SL McEvoy CR Kidd M Beyers N Victor TC van Helden PD Warren RM A recently evolved sublineage of the Mycobacterium tuberculosis Beijing strain family is associated with an increased ability to spread and cause disease J Clin Microbiol 2007 45 1483 1490 17360841 10.1128/JCM.02191-06 PMC1865897

[B35] Cowley D Govender D February B Wolfe M Steyn L Evans J Wilkinson RJ Nicol MP Recent and rapid emergence of W-Beijing strains of Mycobacterium tuberculosis in Cape Town, South Africa Clin Infect Dis 2008 47 1252 1259 18834315 10.1086/592575

[B36] Hirsh AE Tsolaki AG DeRiemer K Feldman MW Small PM Stable association between strains of Mycobacterium tuberculosis and their human host populations Proc Natl Acad Sci USA 2004 101 4871 4876 15041743 10.1073/pnas.0305627101 PMC387341

[B37] Strategic Plan for the Campaign against the Bovine Tuberculosis in Mexico, 2008–2012 [in Spanish] [http://www.senasica.gob.mx/includes/asp/download.asp?IdDocumento=2183&IdUrl=4809]

[B38] Corner LA Nicolacopoulos C Comparison of media used for the primary isolation of Mycobacterium bovis by veterinary and medical diagnostic laboratories Aust Vet J 1988 65 202 205 3048238 10.1111/j.1751-0813.1988.tb14457.x

[B39] Allix-Beguec C Harmsen D Weniger T Supply P Niemann S Evaluation and strategy for use of MIRU-VNTRplus, a multifunctional database for online analysis of genotyping data and phylogenetic identification of Mycobacterium tuberculosis complex isolates J Clin Microbiol 2008 46 2692 2699 18550737 10.1128/JCM.00540-08 PMC2519508

[B40] Cosivi O Grange JM Daborn CJ Raviglione MC Fujikura T Cousins D Robinson RA Huchzermeyer HF de Kantor I Meslin FX Zoonotic tuberculosis due to Mycobacterium bovis in developing countries Emerg Infect Dis 1998 4 59 70 9452399 10.3201/eid0401.980108 PMC2627667

